# The Food We Eat

**Published:** 1854-01

**Authors:** 


### THE FOOD WE EAT.

Hufeland calls the stomach *Atria mortis*, the entrance-hall of death, and says without a good stomach it is impossible to attain a great age. All have naturally good stomachs, that is, good digestion, but it is ruined early by improper feeding as to time, quality, quantity, and mode of preparation. Therefore a large portion of the *Journal* will be occupied with statements in reference to these items. Substantial, nourishing food, properly prepared and well digested, these three are the great essentials of a long and healthy life. I will here give two examples, full of instruction, highly encouraging and well worthy of imitation by all who would like to live in the full enjoyment of health, and all the faculties of mind and body, for a hundred or hundred and fifty years or more. The first shows how an injured stomach and constitution may be repaired, the second how they may remain in perfect health for a hundred and fifty years, and all by the proper management of eating and drinking.

Lewis Cornaro, an Italian nobleman of wealth, by intemperance and debauchery made a wreck and ruin of his fortune and constitution at the early age of forty years. His physicians considering his habits inveterate, informed him that restoration was impossible, and with characteristic recklessness he resolved that if he had to die, he would abandon himself to the fullest indulgences, and thus get all the good possible out of the short remnant of life before him. But some circumstance shortly occurred which induced him to reverse his decision, and experiment on the possibility of disappointing his doctors and his heirs, and living to a good old age. This he attempted at once by means of his food and drink alone. He began by eating and drinking very little, and found that his health improved. Sometimes he would eat more, then less, until he discovered what amount of food was most suitable for him, which was twelve ounces of solid food, and thirteen ounces of fluid, every twenty-four hours. At length his health became so good, that his friends suggested to him, that now he was so hearty and well, there was no longer any necessity for such a strict allowance, and that if he ate and drank a little more it would be of advantage to him. He replied that he was now well, and had continued well for some years, on this allowance, that he could not be better, and that he had no disposition to run any unnecessary risks, nor to make hazardous experiments, and that as he had regained his title and estates, and his health too, he now wished greatly to preserve the last, that he might long enjoy the others. However, he was at length induced to gratify his friends, and increased his food to fourteen, and his drink to sixteen ounces a day, and said he,

"Scarcely had I continued this mode of living ten days, when I began, instead of being cheerful and lively as before, to become uneasy and dejected, a burden to myself and to others. On the twelfth day I was seized with a fever of such violence, for thirty-five days, that my life was despaired of. But by the blessing of God, and my former regimen, I recovered; and now in my eighty-third year, I enjoy a happy state of both body and mind. I can mount my horse unaided. I climb steep hills. When I return home from a private company, or the senate, I find eleven grand-children, whose education, amusements and songs are the delight of my old age. I myself often sing with them, for my voice is clearer and stronger than it was in my youth; and I am a stranger to those peevish and morose humors which so often fall to the lot of old age."

